# Retinal Degeneration and Microglial Dynamics in Mature Progranulin-Deficient Mice

**DOI:** 10.3390/ijms222111557

**Published:** 2021-10-26

**Authors:** Kei Takahashi, Shinsuke Nakamura, Masamitsu Shimazawa, Hideaki Hara

**Affiliations:** Molecular Pharmacology, Department of Biofunctional Evaluation, Gifu Pharmaceutical University, 1-25-4 Daigaku-nishi, Gifu 501-1196, Japan; takahashi.yakkou@gmail.com (K.T.); nakamuras@gifu-pu.ac.jp (S.N.); shimazawa@gifu-pu.ac.jp (M.S.)

**Keywords:** progranulin, retina, retinal pigment epithelium, microglia, lysosome, neuronal ceroid lipofuscinosis

## Abstract

Progranulin (PGRN) is a secreted glycoprotein that regulates numerous cellular processes. The role of PGRN as a regulator of lysosomes has recently received attention. The purpose of this study was to characterize the retinal phenotype in mature PGRN knockout (*Grn*^−/−^) mice. The a-wave amplitude of scotopic electroretinogram and outer nuclear thickness were significantly reduced at 6 months of age in *Grn^−/−^* mice compared to wild-type (*Grn^+/+^*) mice. In *Grn^−/−^* mice, retinal microglial cells accumulated on the retinal pigment epithelium (RPE) apical layer, and the number of infiltrated microglia and white fundus lesions between 2 and 6 months of age showed a close affinity. In *Grn^+/+^* mice, PGRN was located in the retina, while the strongest PGRN signals were detected in the RPE-choroid. The different effects of PGRN deficiency on the expression of lysosomal proteins between the retina and RPE-choroid were demonstrated. Our data suggest that the subretinal translocation of microglia is a characteristic phenotype in the retina of mature PGRN knockout mice. The different effects of PGRN deficiency on the expression of lysosomal proteins between the retina and RPE-choroid might modulate microglial dynamics in PGRN knockout mice.

## 1. Introduction

Progranulin (PGRN) is a secreted glycoprotein by neuron and immune cells, which has been reported to regulate numerous cellular processes, including cell survival, inflammation, and protein clearance [1]. In particular, PGRN has received attention as a regulator of lysosomal function. Evidence supporting a role for PGRN in the lysosome includes its localization to these bodies, the fact that PGRN-deficient mice demonstrate increased reactivity to some lysosomal proteins, as well as its influence on the acidification of lysosomes [2,3]. These results support the role of PGRN in regulating the formation and function of lysosomes.

Haploinsufficiency of the PGRN is a leading cause of frontotemporal degeneration (FTD) due to heterozygous mutations in the granulin (*Grn*) gene. Patients with homozygous *Grn* mutations exhibit adult-onset neuronal ceroid lipofuscinosis (NCL), a lysosomal storage disease. Clinically, both FTD and NCL patients display retinal degeneration and visual dysfunction [4,5]. In addition, both heterozygous and homozygous mutations in the *GRN* gene are associated with autofluorescent aggregates in the retina and brain [6,7]. Characterizing the ocular phenotype in lysosomal storage disorders is helpful in understanding the molecular mechanisms and could also be useful in developing diagnostic tools.

There are at least 14 different variants of NCL, each caused by mutations in different genes. The causative genes of the several distinct NCL subtypes are called CLNs (CLN1-CLN8 and CLN10-CLN14). Transgenic or naturally occurring mouse models that show characteristic retinal phenotypes are available for most CLNs [8,9,10,11,12]. In addition, various mouse models of PGRN deficiency have been generated for CLN11, which is caused by homozygous *Grn* gene mutations, and retinal degeneration and the deposition of autofluorescent aggregates have been characterized in these mice [6,13,14]. In our previous studies, we demonstrated the degeneration of photoreceptors and retinal ganglion cells and abnormal astrogliosis in the retina of young PGRN-deficient mice [15,16]. However, the retinal features of mature PGRN-deficient mice have not been completely characterized. In the present study, we demonstrated, using mature PGRN-deficient mice and age-matched wild-type mice, the characteristic fundus features, progressive retinal degeneration, and the different effects of PGRN deficiency between the retina and RPE-choroid in PGRN-deficient mice. Specifically, we focused on the relationship between fundus lesions and microglial dynamics. Our findings provide novel insights into the role of PGRN in the retina and RPE-choroid.

## 2. Results

### 2.1. Photoreceptor Degeneration in PGRN-Deficient Mouse Retina

**Figure 1:**
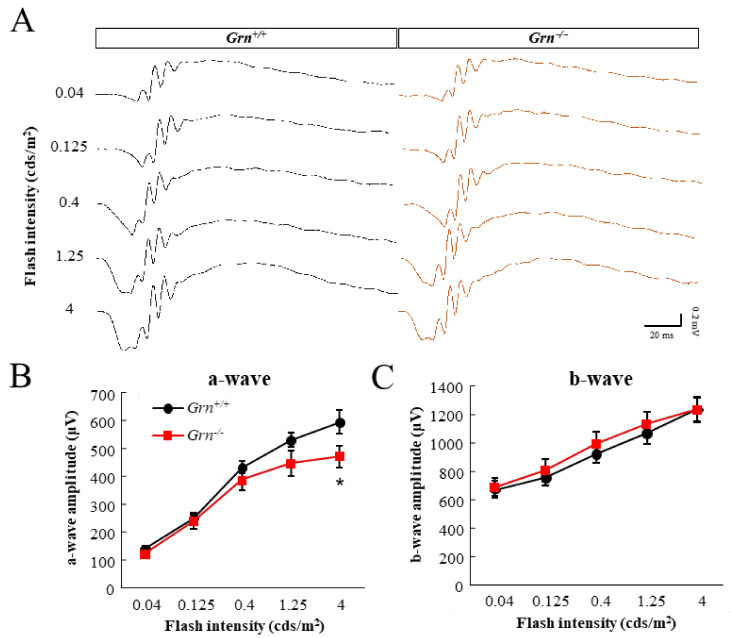

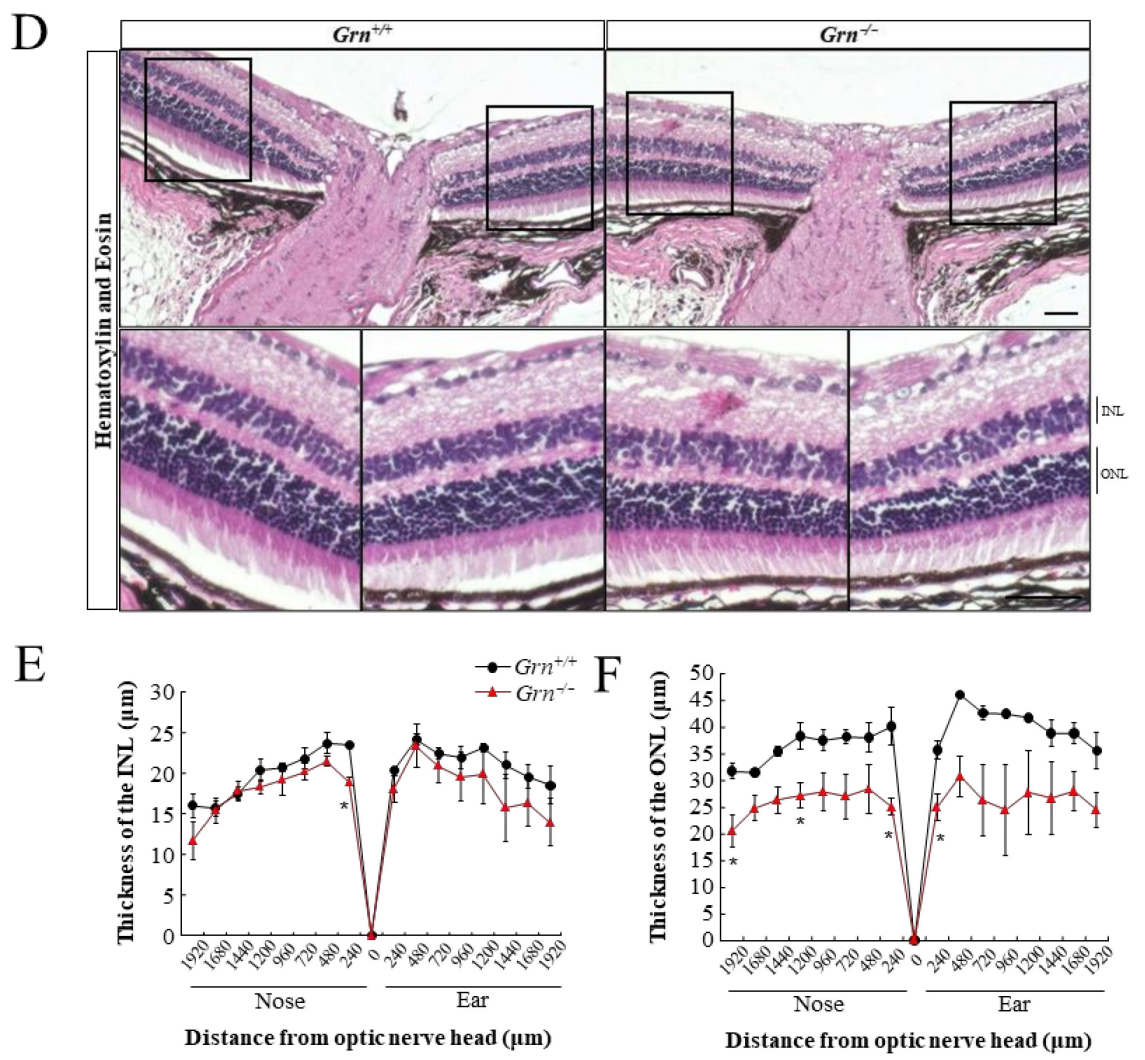
Scotopic-electroretinograms and retinal histology of 6-month-old PGRN-deficient mice. (**A**) Representative dark-adapted ERG responses of the a- and b-wave recorded at 6 months of age. (**B**,**C**) Mean amplitudes of a- and b-waves of the ERGs of *Grn^+/+^* and *Grn^−/−^* mice (*Grn^+/+^*, *n* = 9; *Grn^−/−^*, *n* = 13). (**D**) Representative images of hematoxylin and eosin stained retinal sections of *Grn^+/+^* and *Grn^−/−^* mice. ONL, outer nuclear layer; INL, inner nuclear layer. Scale bar, 50 µm. (**E**,**F**) The mean thickness of INL and ONL (*Grn^+/+^*, *n* = 3; *Grn^−/−^*, *n* = 3). Data are the means ± standard error of the means (SEMs). * *p* < 0.05 vs. *Grn^+/+^* mice (Welch’s *t-tests*).

To examine the effects of PGRN deficiency on retinal cell functions, scotopic-electroretinograms (ERGs) were recorded in 6-month-old *Grn*-knockout (*Grn^−/−^*) mice and *Grn* WT (*Grn^+/+^*) C57BL/6J mice. The ERG a-wave amplitude was significantly smaller in *Grn^−/−^* mice than in *Grn^+/+^* mice at 6 months of age. In contrast, the b-wave amplitude was normal in *Grn^−/−^* mice (Figure 1A–C). Histological inspection of retinal sections confirmed that the outer nuclear layer (ONL) was affected in 6-month-old *Grn^−/−^* mice. The inner nuclear layer (INL) thickness of *Grn^−/−^* mice appeared to be reduced in some regions, while reductions in most areas were not significant (Figure 1D,E). Instead, the ONL thickness was significantly thinner in *Grn^−/−^* mice than in *Grn^+/+^* mice (Figure 1D,F).

### 2.2. Subretinal Deposits in PGRN-Deficient Mouse

**Figure 2:**
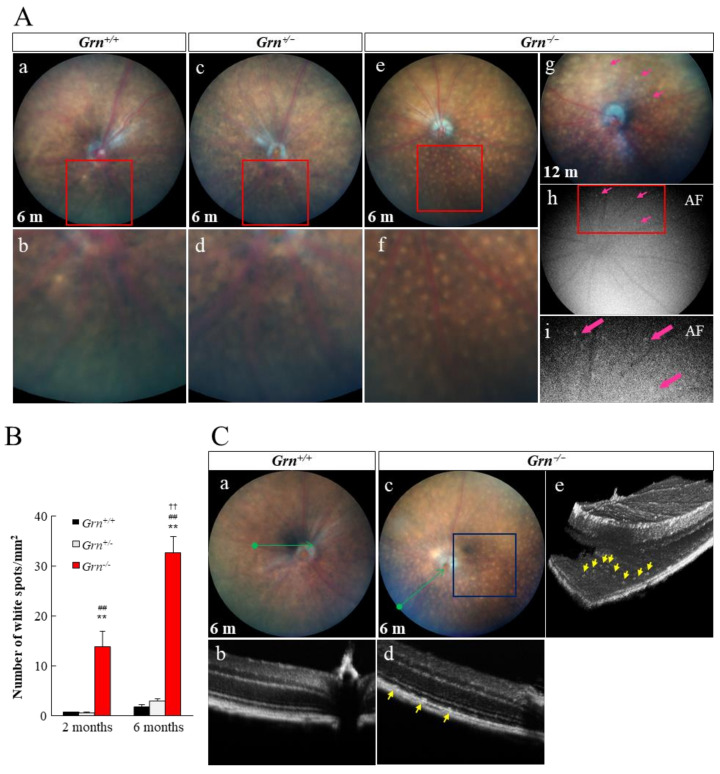
Characteristic fundus lesions and subretinal deposits in *Grn^−/−^* mice. (**A**) Representative fundus color images were taken from 6-month-old (**a**) *Grn^+/+^*, (**c**) *Grn^+/−^*, and (**e**) *Grn^−/−^* mice. (**b**,**d**,**f**) are the zoomed images. The autofluorescent aggregates in 12-month-old (**g**) *Grn^−/−^* fundus were observed with an exposure of high intensity blue light (**h**,**i**). The aggregates are shown with pink arrows. The boxed regions are enlarged in the bottom rows, respectively. (**B**) Quantitative analysis of the number of fundus white lesions in *Grn^+/+^*, *Grn^+/−^*, and *Grn^−/−^* mice at 2 and 6 months of age (2-month-old *Grn^+/+^*, *n* = 3; 2-month-old *Grn^+/−^*, *n* = 6; 2-month-old *Grn^−/−^*, *n* = 4; 6-month-old *Grn^+/+^*, *n* = 6; 6-month-old *Grn^+/−^*, *n* = 6; 6-month-old *Grn^−/−^*, *n* = 6). Data are the means ± SEMs. ** * p* < 0.01 vs. *Grn^+/+^*, ^##^ *p* < 0.01 vs. *Grn^+/−^,* ^††^ *p* < 0.01 vs. 2-month-old *Grn^−/−^* (one-way ANOVA followed by Tukey’s HSD test). (**C**) Cross-sectional (**b**,**d**) and three-dimensional (**e**) optical coherence tomography images from 6-month-old (**a**) *Grn^+/+^* and (**c**) *Grn^−/−^* mice. Green arrowheads indicate the region of cross-sectional images. Blue square represents the region of the three-dimensional image. Subretinal deposits just above the RPE layer in PGRN-deficient mice are shown with yellow arrows.

Representative fundus color images were taken from 2-, 6-, and 12-month-old *Grn^−/−^* mice and compared to those of age-matched *Grn^+/+^* and *Grn^+/−^* mice (Figure 2A and Supplementary Figure S1). *Grn^+/+^* and *Grn^+/−^* mice displayed a healthy fundus up to six months of age. *Grn^−/−^* mice showed accumulation of white fundus deposits at 2 months of age, and the number of deposits increased further from 2 to 6 months of age (Figure 2B). The autofluorescent storage materials were observed at the same location as the white fundus deposits when excited with light at a wavelength of 488 nm (Figure 2Ag–Ai). Cross-sectional and three-dimensional optical coherence tomography (OCT) images showed subretinal deposits in the RPE layer in *Grn^−/−^* mice (Figure 2C).

### 2.3. Infiltration of Retinal Microglia into Subretinal Area in PGRN-Deficient Mouse

**Figure 3:**
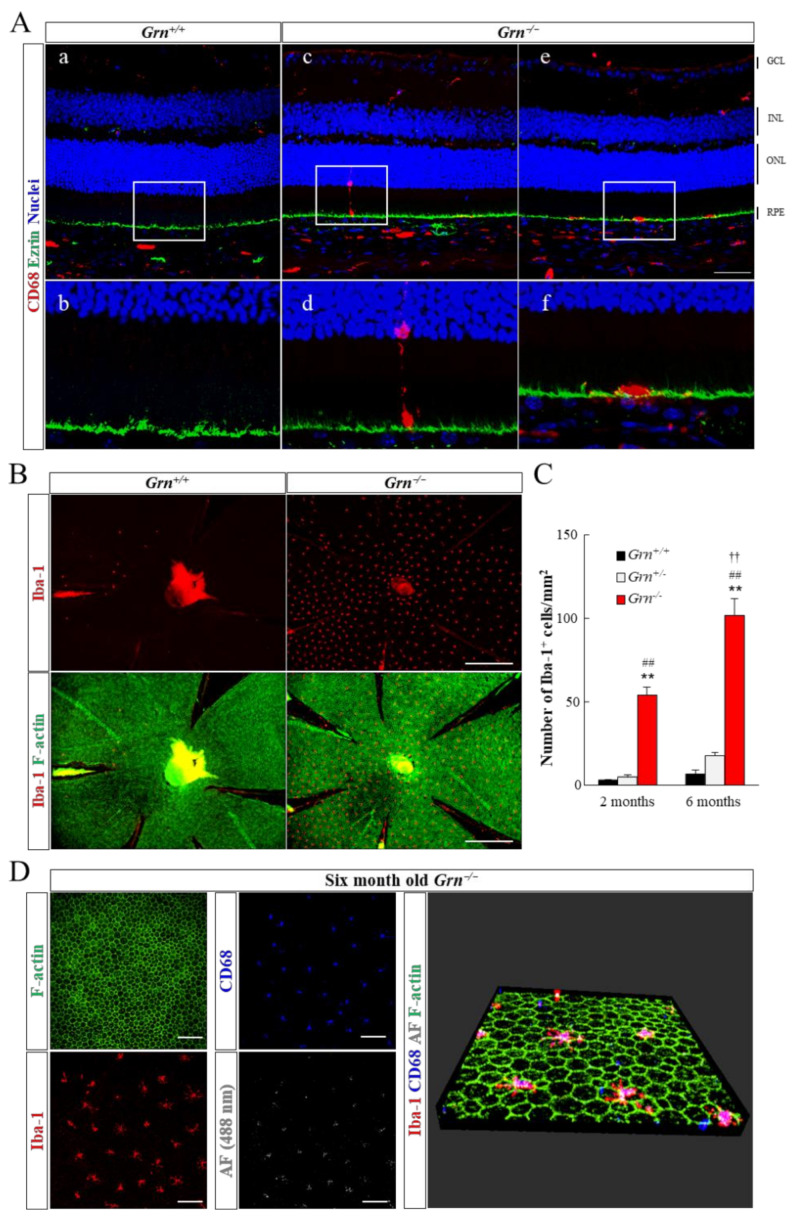
Infiltration and accumulation of retinal microglia into the RPE apical side in *Grn^−/−^* mice. (**A**) The dynamics of retinal microglia in PGRN-deficient retina were assessed with immunostaining for CD68 (red) and ezrin (green) of retinal cross-sections from *Grn^+/+^* (**a**,**b**) and *Grn^−/−^* (**c**–**f**) mice (6 months old). Nuclei were stained with Hoechst 33342 (blue). The boxed regions are enlarged in the second rows, respectively. GCL, ganglion cell layer; INL, inner nuclear layer; ONL, outer nuclear layer; RPE, retinal pigment epithelium. Scale bars: 50 μm. (**B**) Representative microscopic images were the RPE apical side of 6-month-old *Grn^+/+^* and *Grn^−/−^* mice. Immunostaining for Iba-1 (red) and F-actin (green) staining with fluorescent-labeled phalloidin were performed. Scale bars: 500 μm. (**C**) Quantitative analysis of the number of Iba-1-positive cells adjacent to RPE in *Grn^+/+^*, *Grn^+/−^*, and *Grn^−/−^* mice at 2 and 6 month of age (2-month-old *Grn^+/+^*, *n* = 3; 2-month-old *Grn^+/−^*, *n* = 6; 2-month-old *Grn^−/−^*, *n* = 4; 6-month-old *Grn^+/+^*, *n* = 5; 6-month-old *Grn^+/−^*, *n* = 4; 6-month-old *Grn^−/−^*, *n* = 6). Data are the means ± SEMs. ** *p* < 0.01 vs. *Grn^+/+^*, ^##^ *p* < 0.01 vs. *Grn^+/−^,* ^††^ *p* < 0.01 vs. 2-month-old *Grn^−/−^* (one-way ANOVA followed by Tukey’s HSD test). (**D**) Representative images of *Grn^−/−^* RPE flat mount at 6 months of age. Accumulated microglial cells were stained with anti-Iba-1 (red) and anti-CD68 (blue) antibodies. High level of autofluorescence (white) was detected when excited with 488 nm laser. Scale bars: 100 μm.

We detected signs of microglial infiltration into the subretinal area in PGRN-deficient retinal cross-sections and RPE-choroid whole-mount samples (Figure 3). CD68 was predominantly expressed on the lysosomal membranes of microglia and macrophages, while Iba-1 was detected on the plasma membrane of these cells. Therefore, we used CD68 and Iba-1 as the two different microglial markers in this experiment. In 6-month-old *Grn^+/+^* mouse retina, the CD68 signal was restricted to the inner retina (Figure 3Aa,Ab). Simultaneously, some CD68-positive cells infiltrated the ONL in *Grn^−/−^* retinas, and eventually accumulated on the RPE apical layer (Figure 3Ac–Af). The number of Iba-1-positive cells on *Grn^−/−^* RPE was significantly higher than that on *Grn^+/+^* or *Grn^+/−^* RPE at 2 months of age. Moreover, the number of infiltrated microglia on *Grn^−/−^* RPE cells increased further from 2 to 6 months of age (Figure 3B,C). A high level of autofluorescence was detected in accumulated microglia when excited with a 488 nm laser (Figure 3D). 

### 2.4. Expression Pattern of PGRN and Lysosomal Proteins in the Mouse Retina and Retinal Pigment Epithelium-Choroid Complex

**Figure 4:**
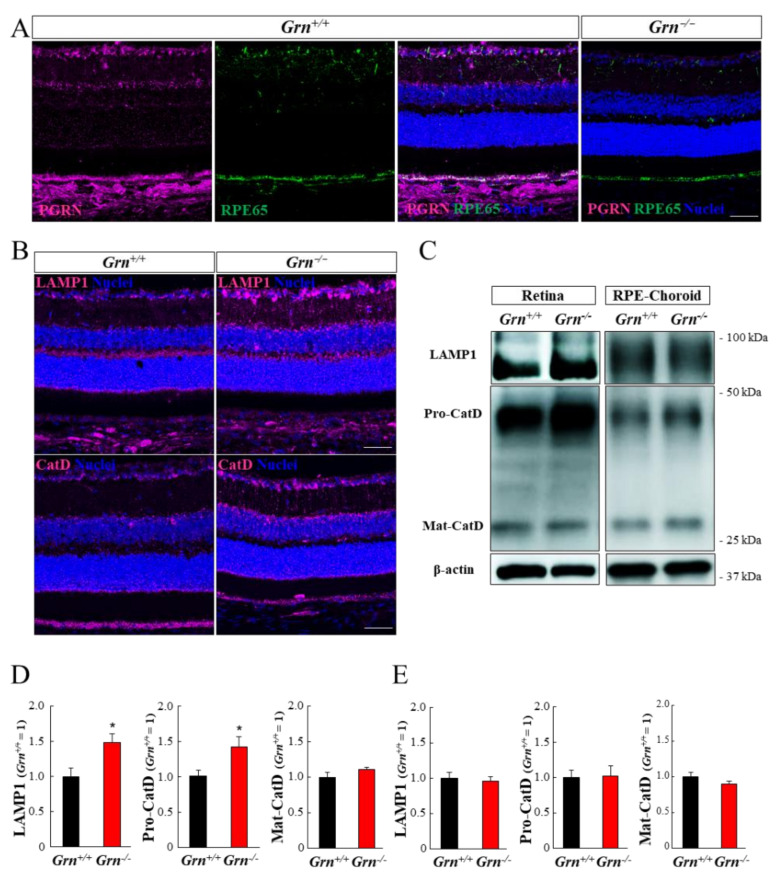
Expression level and location of PGRN and lysosomal proteins in the retina and RPE-choroid complex. (**A**) Representative photographs of immunofluorescence showing PGRN (magenta), RPE65 (green), and Hoechst 33342 (blue). Scale bar, 50 µm. (**B**) The location of LAMP1 and Cathepsin D (magenta) in retinal cross-sections of 6-month-old *Grn^+/+^* and *Grn^−/−^* mice were visualized by immunohistochemistry. Scale bar, 50 µm. (**C**) Representative images of Western blots showing immunoreactivity against LAMP1, cathepsin D, and β-actin. (**D**,E) The expression level of lysosomal proteins in retina (**D**) and RPE-choroid complex (**E**) of *Grn^+/+^* and *Grn^−/−^* mice were assessed by Western blot for LAMP1 and pro- and mature-Cathepsin D at 6 months of age. Data are the means ± SEMs (*Grn^+/+^*, *n* = 4; *Grn^−/−^*, *n* = 5). * *p* < 0.05 vs. *Grn^+/+^* mice (Welch’s *t-tests*).

The expression level and localization of PGRN in the retina and RPE-choroid of *Grn^+/+^* mice were confirmed by immunohistochemistry (Figure 4). PGRN signals were detected in all retinal layers of *Grn^+/+^* mice, except for the photoreceptor inner segment and outer segment (IS/OS) (Figure 4A). The expression level of PGRN was higher in the inner retina, for example, in the ganglion cell layer (GCL) and inner plexiform layer (IPL) than in the outer retina. Moreover, the strongest PGRN signals were detected in the RPE and choroid of *Grn^+/+^* mice. Expression of PGRN could not be observed in any retinal layer or in the RPE-choroid complex of *Grn^−/−^* mice (Figure 4A).

The impact of PGRN deficiency on the expression levels of lysosomal proteins was confirmed by immunohistochemistry and Western blotting at 6 months of age (Figure 4B–E). Immunostaining with retinal cryosections revealed remarkable elevation in the expression levels of lysosomal-associated membrane protein 1 (LAMP1) and lysosomal enzyme cathepsin D (CatD) in mutant retinas (Figure 4B). Conversely, there was no difference in the expression levels of these proteins in the RPE-choroid between *Grn^+/+^* and *Grn^−/−^* mice (Figure 4B). In agreement with the immunohistochemistry data, Western blot analysis revealed a significant increase in LAMP1 and CatD precursor protein (pro-CatD) in the mutant retina, while the expression of these proteins in mutant RPE-choroid did not change (Figure 4C–E). In contrast, the expression level of mature CatD (mat-CatD) remained unchanged between *Grn^+/+^* and *Grn^−/−^* mice in both the retina and RPE-choroid (Figure 4C–E).

### 2.5. Astrogliosis in PGRN-Deficient Mouse Retina

**Figure 5:**
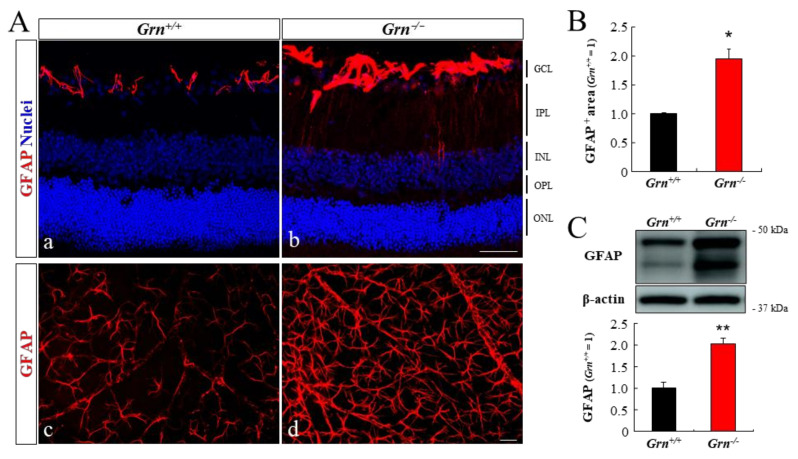
Reactive astrogliosis and Müller cell gliosis in PGRN-deficient mice retinas. (**A**) Expression of GFAP (red) in 6-month-old *Grn^+/+^* and *Grn^−/−^* mice retinas were assessed with immunostaining of retinal cross-sections (**a**,**b**) and retinal flat mounts (**c**,**d**). Nuclei were stained with Hoechst 33342 (blue). GCL, ganglion cell layer; IPL, inner plexiform layer; INL, inner nuclear layer; OPL, outer plexiform layer; ONL, outer nuclear layer. Scale bars, 50 μm. (**B**) Quantitative analysis of the GFAP-positive area in retinal flat mounts (*Grn^+/+^*, *n* = 3; *Grn^−/−^*, *n* = 3). (**C**) The expression level of GFAP in the retina of *Grn^+/+^* and *Grn^−/−^* mice were evaluated by Western blotting (*Grn^+/+^*, *n* = 4; *Grn^−/−^*, *n* = 5). Data are the means ± SEMs. * *p* < 0.05, ** *p* < 0.01 vs. *Grn^+/+^* mice (Welch’s *t-tests*).

Glial fibrillary acidic protein (GFAP) labeling in the retina shows astrocytes and activated Müller glial cells. Expression of GFAP in *Grn^+/+^* retinas was detectable in retinal astrocytes at the age of 6 months (Figure 5Aa). In comparison, GFAP staining in astrocytes was upregulated in *Grn^−/−^* retinal cross-sections (Figure 5Ab). The GFAP-positive area in *Grn^−/−^* retinal whole mounts was significantly higher than that in the retina of *Grn^+/+^* mice (Figure 5Ac,Ad,B). At the same time, the expression level of GFAP in *Grn^−/−^* whole retinas was also elevated when compared with age-matched *Grn^+/+^* mice (Figure 5C).

## 3. Discussion

Retinal degeneration leading to visual dysfunction and blindness has been reported as a typical symptom in patients with FTD and NCL with *GRN* gene mutations [7,17]. In addition, progressive retinal degeneration and retinal deposits of autofluorescent aggregates were observed in a mouse model of CLN11 disease [6,13,14]. However, precise information regarding the affected functions, regions, and cell types in the adult retina with PGRN deficiency is not yet available. In the present study, we analyzed the retinal phenotype of PGRN knockout mice (*Grn^−/−^*) in detail. 

In measurements of retinal function, we found that the scotopic a-wave of ERG was selectively attenuated in amplitude in PGRN-deficient mice at 6 months of age, while the b-wave amplitude showed no difference between *Grn^−/−^* and *Grn^+/+^* mice (Figure 1A–C). Following a brief flash of light from darkness, the scotopic ERG a-wave is generated by rod photoreceptor currents, and b-waves are derived from depolarizing bipolar cell currents [18]. Therefore, attenuation of the a-wave indicates photoreceptor cell dysfunction. In an analysis of retinal histology in age-matched mice, the ONL thickness of *Grn^−/−^* mice was significantly thinner than that of *Grn^+/+^* mice, while the INL thickness of *Grn^−/−^* mice was slightly affected (Figure 1D–F). In our previous reports, we showed that the phenotypes of photoreceptor degeneration, including the thinning of ONL and the decreased expression level of rhodopsin, already appear in 2–3-months-old *Grn^−/−^* mice [15]. Moreover, exogenous PGRN has been shown to have protective effects on attenuated photoreceptors [19,20]. These results indicate that PGRN is necessary to maintain the function and structure of photoreceptor cells. 

The most interesting findings in this study are the relationships between infiltrated microglia in the subretinal area and white fundus lesions in *Grn^−/−^* mice. The rate of increase in the number of migrated microglia into the RPE apical layer and white fundus lesions between 2 and 6 months of age have a close affinity. From cross-sectional and three-dimensional OCT images, the white materials in the fundus images were presumed to be located in the subretinal region. Moreover, both accumulated microglia and fundus spots emit autofluorescence, and the locations of these materials are matched (Figure 2 and Figure 3). Based on these results, it could be suggested that mis-located microglia on *Grn^−/−^* RPE were detected as white fundus lesions and subretinal hyperreflective deposits in OCT images. In the healthy retina, retinal microglial cells are predominantly located in the inner retina, including the GCL, IPL, and outer plexiform layer, and are not present in the subretinal area [21]. Previous studies have demonstrated that retinal microglia are activated and translocated into the subretinal space during healthy aging and retinal disease processes, including age-related macular degeneration and retinitis pigmentosa [22,23,24]. Abnormal activation of microglia and fundus lesions have been observed in multiple mouse models of retinal disease, including NCL [25,26,27,28]. Some studies have demonstrated that pharmacological modulation or ablation of subretinal microglia leads to attenuated photoreceptor degeneration in mouse models of retinal degeneration [29]. Although infiltration and activation of microglia in the subretinal space are common phenotypes in various retinal degenerative diseases, the detailed mechanisms have not been elucidated. A detailed study of the relationship between PGRN, retinal microglia, and RPE may thus help to understand these mechanisms.

The expression levels of LAMP1 and pro-CatD were increased in the inner retina, where PGRN was highly expressed in wild-type mice (Figure 4). Cathepsin D is a lysosomal protease which requires cleavage steps from an inactive precursor (pro-CatD) to the mature state (mat-CatD) by the other lysosomal protease including Cathepsin L and Cathepsin B [30]. These enzymes have optimal activity at acidic pH. It has been reported that PGRN is involved in the process of lysosomal acidification [3]. The result that the expression level of mat-CatD did not increase in *Grn^−/−^* retina might indicate that the PGRN deficiency suppresses the acidification of lysosome in retinal tissue. Moreover, an increase in the number of microglia and excessive Müller and astrogliosis was observed in the inner retina of *Grn^−/−^* mice (Supplementary Figure S2 and Figure 5). In the central nervous system, PGRN is mainly expressed in microglial cells and neurons [31]. Loss of PGRN has been reported to lead to lysosomal dysfunction and overexpression of lysosome-related proteins in neurons and microglia [3,32,33]. In addition, PGRN-deficient microglia exhibit pro-inflammatory phenotypes and induce abnormal activation of astrocytes in the brain [1,34,35]. Therefore, PGRN in the retina might also play important roles in the maintenance of lysosomal function and homeostatic balance in these cells. In other mouse models of CNLs, similar phenotypes, including dysregulation of lysosomes and excessive gliosis in the inner retina, have been reported [8,11,12]. In contrast, the expression of lysosomal proteins in the RPE-choroid was not affected by PGRN deficiency, while the highest expression of PGRN was observed in the RPE-choroid complex in the ocular sections of *Grn^+/+^* mice (Figure 4). The RPE plays an important role in the clearance of the photoreceptor OS to maintain homeostasis of the outer retina. Lysosomes are indispensable in the intracellular degradation of RPE cells, including heterophagy and autophagy [36]. In a previous study, we demonstrated that the addition of recombinant PGRN to human RPE cell culture promoted the phagocytic activity of RPE cells [37]. Based on these findings, it might be reasonable to assume that the lysosome regulator PGRN is highly expressed in the RPE layer and may play a central role in the regulation of intercellular digestion of RPE. However, few studies have focused on the morphological and functional changes in RPE in CLN models [38]. Thus, future studies are needed to elucidate the exact functions of PGRN in RPE cells. 

In summary, our data suggest that subretinal translocation of microglia is a characteristic phenotype in the retina of mature PGRN knockout mice. The different effects of PGRN deficiency on the expression of lysosomal proteins between the retina and RPE-choroid might affect microglial dynamics in PGRN knockout mice. However, it is important to highlight that the small sample size in each experiment is one of the limitations of present study. Detailed knowledge of the progression of photoreceptor degeneration and microglial dynamics in progranulin-deficient mice at the cellular and molecular levels might help to disentangle the pathological basis of NCL and other retinal degenerative diseases.

## 4. Materials and Methods

### 4.1. Animals

*Grn*^−/−^ mice generated by Kayasuga et al. [39] were obtained from the Riken BioResource Center (Tsukuba, Japan) and backcrossed with C57BL/6J mice (Charles River Laboratories Japan, Yokohama, Japan). Genotyping was performed as described in the data sheet provided by the Riken BioResource Center. All mice were housed in temperature-controlled room maintained at 22 ± 2 °C under a 12:12 h light/dark cycle. The mice had free access to a standard diet (CLEA Japan, Tokyo, Japan) and tap water. The number of mice used for each experiment is described in the corresponding figure legends. All investigations were performed in accordance with the Association for Research in Vision and Ophthalmology (ARVO) Statement on the Use of Animals in Ophthalmic and Vision Research. The protocols for all animal experiments were approved by the Animal Experimental Committee of Gifu Pharmaceutical University (approval number. 2017-072 [19-6-2017~28-3-2019] and 2020-050 [22-6-2020~20-3-2021]).

### 4.2. Fundus Photography and Optical Coherence Tomography

The retinal structures were visualized using the Micron IV imaging system (Phoenix Research Laboratories, Pleasanton, CA, USA) and an OCT scan head equipped with a mouse objective lens (Phoenix Research Laboratories). The mice were anesthetized with a mixture of ketamine (80 mg/kg; Daiichi-Sankyo, Tokyo, Japan) and xylazine (6 mg/kg; Bayer Yakuhin, Osaka, Japan), and the pupils were dilated with mydriasis containing 1% tropicamide and 2.5% phenylephrine (Santen Pharmaceutical, Osaka, Japan). Hydroxyl ethyl cellulose (Santen Pharmaceutical) was used to prevent dehydration of the cornea. The mice were placed on the bracket, and the optical lens (light source wavelength 830 nm) was moved close to the cornea along the visual axis. Fundus color photographs and OCT images were collected synchronously in real-time. Fundus monochrome photographs were taken with a 488 nm light.

### 4.3. Scotopic-Electroretinograms

Dark-adapted electroretinograms (ERGs) were recorded at 6 months of age. Mice were placed in a dark room for 24 h before ERG recordings, and subsequently anesthetized with an intraperitoneal injection of a mixture of ketamine (80 mg/kg) and xylazine (6 mg/kg). The pupils were dilated with 5 μL of 1% tropicamide and 2.5% phenylephrine (Santen Pharmaceutical). Flash ERGs were recorded (Power Lab/8SP and LabChart software; AD Instruments, New South Wales, Australia) from the left eye of the mice. The ERGs were recorded with a golden ring corneal electrode (Mayo, Aichi, Japan) and a reference electrode on the tongue. A needle was inserted subcutaneously near the tail of the ground electrode. During the ERG recordings, the mice were kept on a heating pad to maintain a constant body temperature. Light flashes were delivered using a hemisphere stimulator (Mayo). The ERGs were elicited by stimuli of 0.04, 0.125, 0.4, 1.25, and 4.0 cds/m^2^. The digital band-pass filters were set at 0.3 to 500 Hz to isolate the a- and b-waves. The a-wave amplitude was measured from the baseline to the trough of the a-wave, while the b-wave was measured from the trough of the a-wave to the highest b-wave peak.

### 4.4. Histological Analyses

The enucleated eyes were fixed in 4% paraformaldehyde (PFA) for 24 h at 4 °C and then embedded in paraffin. Three paraffin-embedded sections (5 μm) were cut through the optic disc of each eye, prepared in the standard manner, and stained with hematoxylin and eosin. The sections were examined and photographed using an all-in-one fluorescence microscope (BZ-X710; Keyence, Osaka, Japan), and the thickness of the inner nuclear layer (INL) and the ONL were measured at 240 μm intervals from the optic disc to the periphery in the photographs with ImageJ software (National Institutes of Health, Bethesda, MD, USA).

### 4.5. Immunohistochemistry of Ocular Sections and Flatmount

For cryosection preparation, the eyes were enucleated after cervical dislocation, fixed in 4% PFA for at least 24 h at 4 °C, and then immersed in 25% sucrose in 0.01 M phosphate buffered saline (PBS) for 2 days. The eyes were then embedded in optimal cutting temperature compound (Sakura Finetek Japan, Tokyo, Japan) and flash frozen in liquid nitrogen. Fifteen micrometer sections were cut with a cryostat and mounted on glass slides. The eyes for flatmount preparation were enucleated and fixed in 4% PFA for 12 h. After removing the cornea and lens, the retina and the RPE-choroid-sclera complex were isolated. The samples were blocked with 10% non-immune horse serum (Vector Laboratories, Burlingame, CA, USA) containing 0.3% Triton X-100 (Bio-Rad, Hercules, CA, USA, catalog 161-0407) for 1 h, and then incubated with the primary antibody at 4 °C overnight. The next morning, the samples were covered with a secondary antibody for 1 h and then counterstained with Hoechst 33342 (1:1000; Invitrogen, Waltham, MA, USA, catalog H3570) for 15 min.

The following primary antibodies were used: sheep anti-mPGRN (1:100; R&D Systems, Minneapolis, MN, USA, catalog AF2557), mouse anti-RPE65 (1:100; Abcam, Cambridge, MA, UK, catalog ab13826), rat anti-LAMP1 (1:200; Abcam, catalog ab25245), goat anti-cathepsin D (1:200; R&D systems, catalog AF1029), rabbit anti-GFAP (1:500; SHIMA Laboratories, Tokyo, Japan, catalog ROI003), rabbit anti-Iba1 (1:200; FUJIFILM Wako Pure Chemicals, Osaka, Japan, catalog 019-19741), mouse anti-ezrin (1:100; Santa Cruz Biotechnology, Dallas, TX, USA, catalog sc-58758), and rat anti-CD68 (1:200; Bio-Rad, catalog MCA1957GA). The following secondary antibodies were used: Alexa Fluor^®^ 647 donkey anti-sheep IgG (1:1000; Invitrogen, catalog A21448), Alexa Fluor^®^ 488 donkey anti-mouse IgG (1:1000; Invitrogen, catalog A32766), Alexa Fluor^®^ 546 donkey anti-rabbit IgG (1:1000; Invitrogen, catalog A10040), Alexa Fluor^®^ 647 donkey anti-rat IgG (1:1000; Jackson ImmunoResearch, West Grove, PA, USA, catalog 712-605-153), and Alexa Fluor^®^ 647 donkey anti-goat IgG (1:1000; Jackson ImmunoResearch, catalog 705-605-003). The stained sections and flatmounts were photographed using a confocal microscope (FLUOVIEW FV3000; Olympus, Tokyo, Japan).

### 4.6. Western Blot Analysis

For Western blot analyses, the eyes were enucleated after cervical dislocation, and the retinas and RPE-choroid-sclera complexes were isolated and flash frozen in liquid nitrogen. To extract proteins, the tissue was homogenized in RIPA buffer (Sigma-Aldrich, St. Louis, MO, USA, catalog R0278) containing a protease inhibitor and a phosphatase inhibitor cocktail with a homogenizer. The lysate was centrifuged at 12,000 × g for 20 min, and the protein concentration was measured by comparison with known concentrations of BSA with a bicinchoninic acid protein assay kit (Pierce Chemical, Dallas, TX, USA, catalog 23225). 

The protein samples were separated on 5–20% SDS-PAGE gels (FUJIFILM Wako Pure Chemicals, catalog 194-15021), and then transferred onto a polyvinylidene difluoride membrane (Millipore, Billerica, MA, USA, catalog IPVH00010). The membranes were blocked for 1 h at room temperature with Blocking One-P (Nacalai Tesque, Kyoto, Japan, catalog 05999-84), and then incubated with the primary antibody solution at 4 °C overnight. The following primary antibodies were used: rat anti-LAMP1 (1:500; Abcam, catalog ab25245), goat anti-cathepsin D (1:500; R&D Systems, catalog AF1029), rabbit anti-GFAP (1:500; SHIMA Laboratories, catalog ROI003), and mouse anti-β-actin (1:2000; Sigma-Aldrich, catalog A2228). After exposure to the primary antibodies for at least 12 h, the membranes were incubated with horseradish peroxidase (HRP)-conjugated goat anti-rabbit IgG (1:2000; Invitrogen, catalog 31460), rabbit anti-goat IgG (1:2000; Invitrogen, catalog 31402), goat anti-rat IgG (1:2000; Invitrogen, catalog 31470), or goat anti-mouse IgG (1:2000; Invitrogen, catalog 31430) for 1 h at room temperature. The immunoreactive bands were visualized using ImmunoStar LD (FUJIFILM Wako Pure Chemicals, catalog 290-69904), and then measured with the Amersham Imager 680 blot and gel imager (Cytiva, Marlborough, MA, USA).

### 4.7. Statistical Analyses

Data are expressed as the mean ± SEM of at least three independent mice or eyes. Two data sets were compared using a two-tailed Welch’s *t test*. Multiple comparisons were performed using one-way ANOVA followed by Tukey’s honestly significant difference (HSD) test. Statistical significance was set at *p* < 0.05. All statistical analyses were performed using SPSS (version 24.0.0.0; IBM, Armonk, NY, USA).

## Data Availability

Data is contained within the article.

## Supplementary Materials

The following are available online at www.mdpi.com/1422-0067/222/11/1557/s1.
