# Supplementary material for: Retinal Degeneration and Microglial Dynamics in Mature Progranulin-Deficient Mice
Source: Int J Mol Sci. 2021 Oct 26;22(21):11557. doi: 10.3390/ijms222111557 (PMC8584076; doi:10.3390/ijms222111557)
Supplement: Supplementary file 1 [file ijms-22-11557-s001.zip › Supplemenatry_materials.pdf]

**Figure S1: Fundus images of 2 months old mice**

Representative fundus color images were taken from 2 months old *Grn*$^{+/+}$, *Grn*$^{+/-}$, and *Grn*$^{-/-}$ mice.

**Figure S2: Retinal microgliosis in PGRN-deficient mice**

(A) Representative immunofluorescence showing Iba-1 positive cells (red) in 6-month old *Grn*$^{+/+}$ and *Grn*$^{-/-}$ *retinal flat mounts*. Scale bar, 50 μm. (B) Quantitative analysis of the number of Iba-1-positive cells in retinal flat mounts of *Grn*$^{+/+}$ and *Grn*$^{-/-}$ mice at 6 months of age (*Grn*$^{+/+}$, n = 3; *Grn*$^{-/-}$, n = 3). Data are presented as the mean ± SEM. $^{**}P < 0.01$ vs. *Grn*$^{+/+}$ mice (Welch's *t*-tests).
